# Genetic Variants of TPCN2 Associated with Type 2 Diabetes Risk in the Chinese Population

**DOI:** 10.1371/journal.pone.0149614

**Published:** 2016-02-26

**Authors:** Yujuan Fan, Xuesong Li, Yu Zhang, Xiaofang Fan, Ning Zhang, Hui Zheng, Yuping Song, Chunfang Shen, Jiayi Shen, Fengdong Ren, Jialin Yang

**Affiliations:** Department of Endocrinology, Central Hospital of Minhang District, Minhang Hospital affiliated with Fudan University, Shanghai, People’s Republic of China; University of Catanzaro Magna Graecia, ITALY

## Abstract

**Objective:**

The aim of this study was to determine whether TPCN2 genetic variants are associated with type 2 diabetes and to elucidate which variants in TPCN2 confer diabetes susceptibility in the Chinese population.

**Research Design and Methods:**

The sample population included 384 patients with type 2 diabetes and 1468 controls. Anthropometric parameters, glycemic and lipid profiles and insulin resistance were measured. We selected 6 TPCN2 tag single nucleotide polymorphisms (rs35264875, rs267603153, rs267603154, rs3829241, rs1551305, and rs3750965). Genotypes were determined using a Sequenom MassARRAY SNP genotyping system.

**Results:**

Ultimately, we genotyped 3 single nucleotide polymorphisms (rs3750965, rs3829241, and rs1551305) in all individuals. There was a 5.1% higher prevalence of the rs1551305 variant allele in type 2 diabetes individuals (A) compared with wild-type homozygous individuals (G). The AA genotype of rs1551305 was associated with a higher diabetes risk (p<0.05). The distributions of rs3829241 and rs3750965 polymorphisms were not significantly different between the two groups. HOMA-%B of subjects harboring the AA genotype of rs1551305 decreased by 14.87% relative to the GG genotype.

**Conclusions:**

TPCN2 plays a role in metabolic regulation, and the rs1551305 single nucleotide polymorphism is associated with type 2 diabetes risk. Future work will begin to unravel the underlying mechanisms.

## Introduction

Two-pore channel 2 (TPCN2) localizes to the lysosome and is a likely receptor for the calcium-mobilizing agent nicotinic acid adenine dinucleotide phosphate (NAADP)[1]. Several studies have indicated that NAADP may play a role in the insulin signaling of β-cells [2–4] and a recent study suggested its involvement in glucose homeostasis[5]. Another study demonstrated that TPCN2 was differentially expressed in heterogeneous stock rats with glucose intolerance relative to those with normal glucose regulation and demonstrated that TPCN2 expression levels negatively correlated with fasting glucose [6].

All data have pointed to TPCN2 as a new gene contributing to glucose and insulin regulation. Variants within and near TPCN2 have been significantly associated with fasting glucose as well as TPCN2 expression levels in heterogeneous stock rats [6]. Therefore, it is possible that variants within TPCN2 may be associated with diabetes in humans. However, few attempts have been made to examine the extent to which single nucleotide polymorphisms (SNPs) are associated with type 2 diabetes and other human glycemic traits. The purpose of our study was to determine whether the TPCN2 genetic variation is related to type 2 diabetes. We selected 6 SNPs in the TPCN2 gene cluster and examined their status as risk factors for developing type 2 diabetes.

## Research Design and Methods

### Study Subjects

Our study sample consisted of 384 patients diagnosed with type 2 diabetes and 1468 non-diabetic controls. None of the subjects were genetically related to each other. The inclusion criteria for the controls included (1) fasting plasma glucose (FPG) < 6.1 mmol/L; (2) postprandial plasma glucose (PPG) < 7.8 mmol/L; and (3) no personal or family history of diabetes. All participants provided written informed consent before enrollment. The study protocol was approved by the Research Ethics Boards of Minhang Hospital Affiliated with Fudan University and administered in accordance with the Declaration of Helsinki.

### Anthropometric and Clinical Measurements

Anthropometric measurements including height, weight, waist circumference and hip circumference were measured using standardized procedures. Body mass index (BMI) was calculated as weight (kilograms) divided by height (meters) squared. Plasma samples were collected following a 12-hour overnight fast. All samples were run in the same assay. Glucose, triglycerides, high-density lipoprotein, low-density lipoprotein, and total cholesterol were measured on an automatic enzymatic analyzer. HbA_1c_ was determined by standard procedures. Insulin and C-peptide were measured by radioimmunoassay. HOMA-%B and HOMA-IR were calculated using the homeostasis model assessment method.

### SNP Selection, Genotyping and Quality Control (QC) Filters

Several procedures were utilized to select SNPs. First, SNPs and genotypes of the TPCN2 gene were investigated in HapMap-HCB. Second, we calculated the linkage disequilibrium(LD) between SNPs in HapMap-HCB using the Genome Variation Server 138 (http://gvs.gs.washington.edu/GVS138/) and filtered all the monomorphic sites. The allele frequency cutoff was set to 20% and the r^2^ threshold was set to 0.80. Finally, based on previous genetic studies [7,8] as well as public databases NCBI ClinVar database on the TPCN2 gene cluster, 6 candidate tag SNPs (rs35264875, rs267603153, rs267603154, rs3829241, rs1551305, and rs3750965) were selected.

Then, QC filters were applied to SNPs and samples before analysis to ensure robust association tests. We applied the same QC parameters to scans; the following SNPs were excluded: those a missing call rate of ≥2%, more than one discordance, significant deviations from HWE (P<1 × 10^−4^) or a minor allele frequency (MAF) of less than 1%. Three SNPs (rs3750965, rs3829241, and rs1551305) passed the QC criteria and were included ing the analysis.

Extracted DNA of the whole blood genome was analyzed by 1% agarose gel electrophoresis, and the DNA concentration and the degree of DNA degradation were subsequently estimated. Genotypes were determined using a Sequenom MassARRAY SNP genotyping system, which is based on detection through MALDI-TOF MS (Sequenom Inc., San Diego, CA, USA).

### Statistical Analysis

Results are expressed as the mean ± standard deviation (S.D.) or as the ratio. We determined whether each variable was normally distributed prior to statistical testing, and logarithmic transformation was performed on skewed variables. An independent-samples t-test or chi-square test was used to compare the general characteristics and biochemical parameters between the two groups. Analysis of variance (ANOVA) was used to examine quantitative traits among different genotypes; a two-tailed P value < 0.05 was considered statistically significant. These statistical analyses were performed using SPSS (version 17.0; SPSS Inc., Chicago, IL, USA).HWE was tested in the control population using the HWE program (http://ihg.gsf.de/cgi-bin/hw/hwa1.pl). LD was assessed by calculating D′ and r^2^ using Haploview 4.2 (Broad Institute, MA,USA). Power calculations were performed using Quanto v1.2.4. After performing QC, we analyzed the genotypes of 3 SNPs in 384 patients with type 2 diabetes and 1468 controls for association analysis using PLINK software[9].

## Results

### Study Population Characteristics

The anthropometric and biochemistry characteristics of the subjects are presented in Table 1(data in S1 Dataset). The two groups were significantly different with regard to fasting glucose, HbA_1C_, HOMA-%B and HOMA-IR (P < 0.05). BMI, however, was not significantly different between cases and controls (P = 0.109). Likewise, similar proportions of women were present in cases and controls (43.0% and 43.9%, respectively).

**Table 1:** Comparison of clinical and biochemistry characteristics between the two groups.

	Controls	T2DM	P-value
	n = 1468	n = 384	
Age (years)	58.87±14.06	59.58±15.83	0.397
Sex (male/female)	824/644	219/165	0.751
BMI (kg/m^2^)	24.89±3.80	25.28±4.05	0.109
WHR	0.93±0.06	0.94±0.06	0.060
Systolic BP(mmHg)	127.47 ±14.10	128.03±14.79	0.493
Diastolic BP(mmHg)	79.16 ±7.71	79.23±7.90	0.871
FPG (mmol/L)	5.01±0.63	9.03±3.56	0.000*
HbA_1C_ (mmol/mol)	36.12±0.23	90.15±2.71	0.000*
HbA_1C_ (%)	5.49±0.23	10.41±2.71	0.000*
TC (mmol/L)	4.46±1.20	4.58±1.40	0.086
TG (mmol/L)	2.09±2.57	2.19±2.98	0.495
HDL-C (mmol/L)	1.00±0.37	1.01±0.40	0.670
LDL-C (mmol/L)	2.64±0.90	2.70±1.00	0.228
HOMA-%B	78.59±49.91	56.81±47.33	0.000*
HOMA-IR	1.49±0.97	1.75±1.11	0.000*

Notes: Data are presented as the mean ± standard deviation or as the ratio.

*P <0.05.

Abbreviations: BMI, body mass index; WHR, waist-to-hip ratio; BP, blood pressure; FPG, fasting plasma glucose; HbA1c, glycosylated hemoglobin; TC, total cholesterol; TG, triglycerides; HDL-C, high-density lipoprotein cholesterol; LDL-C, low-density lipoprotein cholesterol; HOMA-%B, homeostasis model assessment of steady-state beta cell function; HOMA-IR, homeostasis model assessment of insulin resistance.

### Characteristics of the SNPs

**Fig 1:**
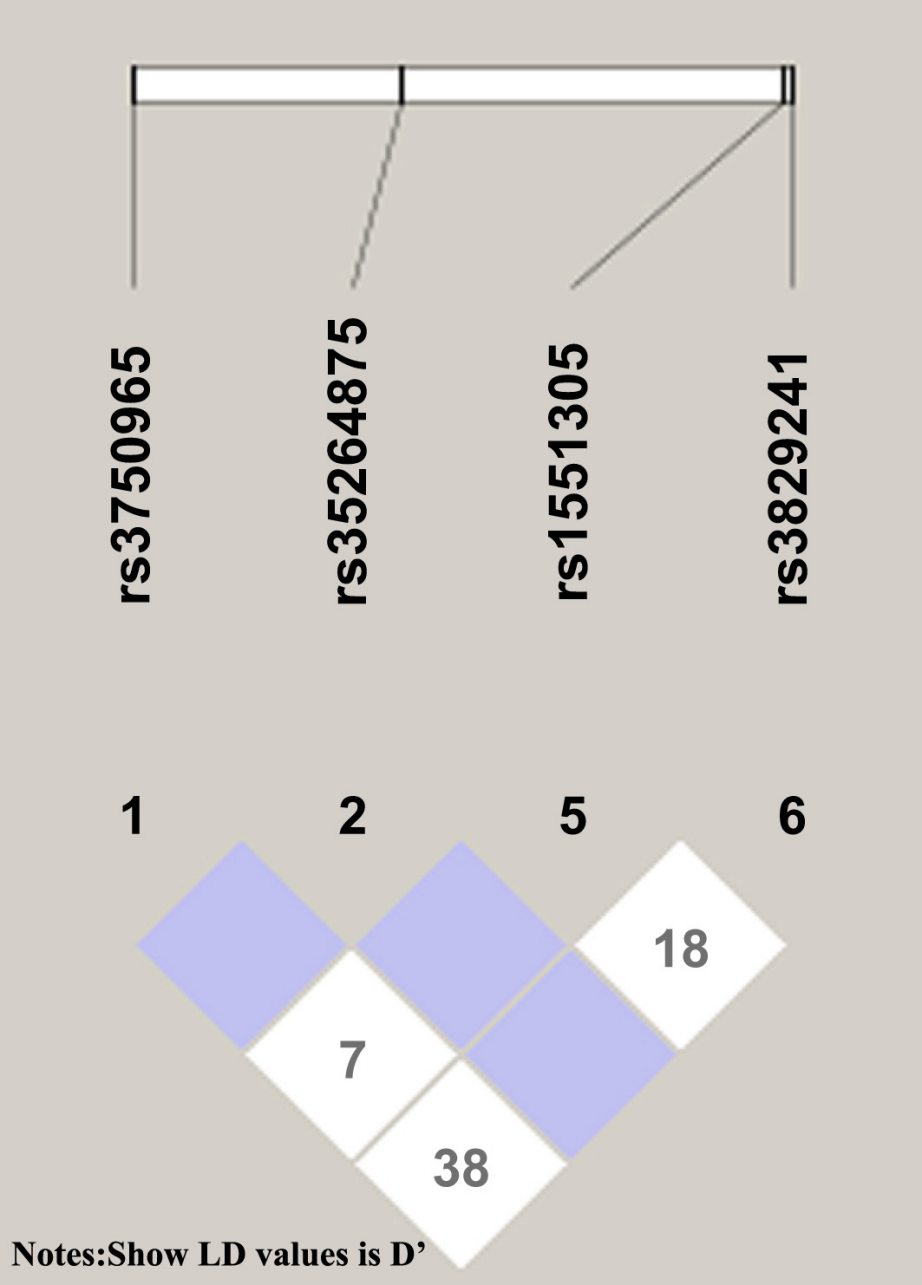
LD between 4 SNPs in the study cohort.

Table 2 presents the characteristics of the SNPs (including their position, genotype frequency, and MAF). The genotyping success rate was 98–99%. All genotype distributions conformed to Hardy–Weinberg equilibrium. SNP LD patterns were assessed using both the D’ and r^2^ values (Fig 1). Rs3750965, rs3829241, rs1551305 and rs35264875 were in weak LD with each other.

**Table 2:** Main characteristics of the 3 SNPs.

SNP	Chromosome	Position	Alleles	GSR(%)	MAF		HWE
					Cases	Controls	
					(n = 384)	(n = 1468)	
rs3829241	11: 69087895	cds	A/G	99.74	0.25	0.24	0.96
rs1551305	11: 69087765	intron	A/G	98.44	0.37	0.32	0.82
rs3750965	11: 69072692	cds	G/A	99.48	0.23	0.25	0.59

Abbreviations: GSR, genotyping success rate; MAF, minor allele frequency; HWE, Hardy-Weinberg equilibrium.

### Allele Frequencies of TPCN2 Polymorphisms and Genotype Distribution

Allele frequencies of TPCN2 polymorphisms are shown in Table 3. There was a 5.1% higher prevalence of the rs1551305 variant allele in type 2 diabetes individuals (A) compared with wild-type homozygote individuals (G). Therefore, carrying the A allele appeared to increase the risk of type 2 diabetes. Table 4 shows the genotype distribution(date in S2 Dataset). The AA genotype of rs1551305 was associated with a higher diabetes risk (P < 0.05). The distributions of the rs3829241 and rs3750965 polymorphisms showed no significant difference between the two groups.

**Table 3:** Allele frequencies of TPCN2 polymorphisms.

SNP	Controls(%)		T2DM (%)		P-value	OR (95% CI)
	A	G	A	G		
rs3829241	748(25.5)	2188(74.5)	174(22.7)	594(77.3)	0.1071	0.86 (0.71–1.03)
rs1551305	913(31.5)	1983(68.5)	276(36.6)	478(63.4)	0.0111*	1.25(1.06–1.48)
rs3750965	2245(76.5)	691(23.5)	575(75.1)	191(24.9)	0.4263	1.08 (0.90–1.30)

Notes: Data are presented as the frequency.

*P <0.05.

**Table 4:** Genotype distributions of the TPCN2 rs3829241, rs1551305 and rs3750965 polymorphisms in controls and T2DM patients.

SNP	Controls (%)			T2DM (%)			P-value
	AA	AG	GG	AA	AG	GG	
rs3829241	95 (6.7)	558 (37.9)	815 (55.4)	19 (6.7)	136 (12.7)	229 (80.6)	0.2712
rs1551305	142 (9.8)	629 (43.4)	677 (46.8)	87 (23.1)	102 (27.1)	188 (49.8)	0.0000^*^
rs3750965	862 (58.7)	521 (35.5)	85 (5.8)	224 (58.5)	127 (33.2)	32 (8.3)	0.1624

Notes: Data are presented as the n(frequency).

*P <0.05.

### Comparison of Quantitative Traits among Three Genotypes of rs1551305

ANOVA showed that the HOMA-%B of subjects harboring the rs1551305 AA genotype was 14.87% less than that for the GG genotype; this difference was statistically significant (P < 0.05). BMI, waist-to-hip ratio, fasting plasma glucose and HbA_1C_ were not significantly different among the three genotypes in the diabetic subjects (Table 5), even when controlling for sex and age.

**Table 5:** Comparisons of quantitative traits among the three rs1551305 genotypes.

		rs1551305		F	P-value
	AA	AG	GG		
BMI (kg/m^2^)	24.82±3.66	25.38±4.28	25.37±4.13	0.538	0.584
WHR	0.92±0.07	0.94±0.06	0.94±0.07	1.757	0.175
FPG (mmol/L)	9.22±3.32	8.48±3.01	9.26±3.92	1.729	0.179
HbA_1c_ (mmol/mol)	92.47±2.52	89.34±3.06	90.25±2.61	0.244	0.785
HbA_1c_ (%)	10.55±2.52	10.33±3.06	10.36±2.61	0.171	0.843
HOMA-%B	52.72±38.62	53.49±46.86	67.59±59.95	3.401	0.034*
HOMA-IR	1.73±1.04	1.77±1.18	1.75±1.13	0.029	0.971

Notes

*P <0.05.

Abbreviations: BMI, body mass index; WHR, waist-to-hip ratio; HbA_1c_, glycosylated hemoglobin; FPG, fasting plasma glucose; HOMA-%B, homeostasis model assessment of steady-state beta cell function; HOMA-IR, homeostasis model assessment of insulin resistance.

## Discussion

Two-pore channels (TPCs) are endolysosomal channels with homologies to TRP (one-domain) and CaV (four-domain) channels, and have a predictable intermediate two-domain structure that probably assembles as dimmers [10,11]. Although a three-gene family, several species, including mice and humans, only have TPCN1 and TPCN2 genes. TPCN2 genes have also been ascribed with metabolic functionality. TPCN2^-/-^ knockout mice have been shown to exhibit significantly decreased insulin response to glucose challenge relative to wild-type mice [6]. These results suggest that TPCN2 may be associated with diabetes. In our study examining the relationship between the genetic variation of rs1551305 in TPCN2 and risk of type 2 diabetes, subjects who harbored the AA genotype of the TPCN2 rs1551305 SNP had a higher incidence of type 2 diabetes. We also found that TPCN2 rs3829241 and rs3750965 variants did not significantly contribute to the incidence of glucose tolerance abnormalities.

Basal insulin secretion, as assessed by HOMA-%B, decreased in subjects harboring the rs1551305 AA genotype. This suggests that the genetic variation of rs1551305 in TPCN2 might be associated with insulin; however, the precise role of TPCN2 with regard to type 2 diabetes risk is still under investigation. The nearby TPCN2 encodes a putative cation-selective ion channel that releases Ca^2+^ from acidic organelles [12]. Pancreatic β cells are electrically excitable and elicit oscillatory bursts of Ca^2+^ action potentials mediated by voltage-dependent Ca^2+^ channels in response to elevated blood glucose concentrations. These responses drive cytosolic Ca^2+^ ([Ca^2+^]i) oscillations which, in turn, induce pulsatile insulin release[13], and defects in their generation may be associated with the loss of glucose homeostasis in type 2 diabetes [14]. Recent studies have suggested that the newly discovered Ca^2+^ mobilizing messenger NAADP might play an important role in ß-cell Ca^2+^ signaling [5, 15–17]. Since the initial reports linking NAADP-evoked Ca^2+^ release to TPCs [18], there have been numerous reports of TPCs playing an essential role in mediating NAADP-evoked Ca^2+^ release from acidic stores[19]. TPCs, as Ca^2+^-permeable channels are indispensable for NAADP signaling, and TPCN2 appears to be the predominant NAADP activated channel [20, 21]. Selective pharmacological inhibition of NAADP-evoked Ca^2+^ release or the genetic ablation of endolysosomal TPCN2 channels attenuates glucose- and sulphonylurea-induced membrane currents, depolarization, cytoplasmic Ca^2+^ signals and insulin secretion. This might be the main reason for the association between the genetic variation of TPCN2 and the risk of type 2 diabetes.

As we know, type 2 diabetes is closely related to obesity. However, no association was observed between TPCN2 polymorphisms and BMI in the diabetes patients in our sample, indicating that the effects of the TPCN2 gene on the development of type 2 diabetes may be independent from the effects of BMI. This finding, however, contrasts with those from previous animal studies. Lear's study, for instance, showed that TPCN1 and TPCN2 double-knockout (Tpcn1/2^(-/-)^) animals had a higher respiratory quotient and became obese between 6 and 9 months of age [22].

TPCN1/2^(-/-)^ mice show mature-onset obesity due to reduced lipid availability and use, as well as a defect in β-adrenergic receptor signaling, leading to impaired thermogenic activity in brown adipose tissue [19]. The lack of an association between TPCN2 polymorphisms and BMI in diabetes patients in this study might be due to the presence of very little brown adipose tissue in adults.

Although no significant associations were found between these SNPs and fasting glucose levels and HbA_1C_, the genotype distributions differed between the diabetic and control groups. This suggests that genetic variation may affect the incidence of diabetes, but not its severity.

In conclusion, our results provide strong evidence that the TPCN2 gene plays a role in metabolic regulation. Genetic variants of rs1551305, but not rs3829241 or rs3750965, are associated with the risk for type 2 diabetes. Future studies should test the hypothesis that TPCN2 is involved in the glucose-stimulated insulin secretion of β-cells.

## Supporting Information

S1 Dataset The anthropometric and biochemistry characteristics of the subjects. (SAV)

S2 Dataset The genotype of 6 SNPs in our study. (XLSX)
